# Proceedings of the Albany County Medical Society

**Published:** 1873-05

**Authors:** F. C. Curtis

**Affiliations:** Secretary


BUFFALO

### Medical and Surgical Journal.

VOL XII. MAY, 1873. No. 10

### Original Communications.

:o:

ART. I.—*Medical Society of the County of Albany. Semi-Monthly Meeting, April* 9, 1873.

Reported by F. C. Curtis, M. D., Secretary.

The President, Dr. Van Derveer, called the meeting to order. After the minutes of the last meeting had been read and approved, the following paper by Dr. James H. Armsby, on Ligature of the Subclavian Artery, was read:

My first operation was at Glen's Falls, in 1863, for axillary aneurism. The patient, aged 28, had lost his right arm by the accidental discharge of a cannon four months previous.

The stump had healed, and was apparently sound, until two months after the accident, when a pulsating and painful tumor formed in the axilla. Its size increased rapidly until the day before I was called, when the sac was ruptured by an effort to put on his coat, and he lost in a few moments two or three quarts of blood. He fainted and was almost pulseless several hours, during which time the opening was closed by compresses. When I arrived I found him in bed, pulse 130, skin cool and bloodless. The aneurismal tumor had filled and was pulsating strongly under the dressings.

I decided to operate immediately. The first incision along the clavicle extended from the mastoid to the trapezius muscle and was about three and one-half inches in length. The second extended vertically, intersecting the first near its middle. In dissecting the flaps, it became necessary to ligate three small arteries, and the external jugular vein, the latter being first secured by two ligatures, between which it was divided. The clavicular portions of the mastoid were divided, and the deep cervical fascia. The clavicle was carried upwards to such an extent by the great size of the tumor as to render it exceedingly difficult to reach the artery. While detaching the fascia and large veins which covered the artery at the scalenus muscle, I was startled by a slight gurgling sound and the presence of air bubbles at the deepest part of the wound, and immediately covered the spot with my finger. There was a tremor and slight convulsive movement, during which the pulse and heart were greatly disturbed, indicating the introduction of a small quantity of air into the circulation. After a moment of extreme anxiety I renewed the operation, and cast the ligature around the artery just under the outer border of the scalenus muscle. The deep cervical and infra-scapular arteries were both exposed and held to one side. They came off on the cardiac side of the scalenus, and as the loss of blood had been so great, I did not deem it safe or necessary to ligate them. The greatest difficulty of the operation consisted in separating the great veins, which completely encircled and obscured the artery. As soon as the ligature was drawn tight, the pulsation in the tumor ceased. An anodyne was administered and the patient had a good night's rest. Under a generous diet and tonic treatment he improved rapidly until the tenth day, when the skin over the tumor became discolored and painful. I was again called and laid open the sac freely, removing nearly a quart of coagula and sanguino-purulent fluid. After this his recovery was rapid and complete. The ligature came away on the twenty-ninth day.

My second operation was for the relief of secondary hemorrhage which threatened an immediate and fatal result. The patient, Major C., of the U. S. Army, was wounded by a minie ball, which entered in front of the shoulder joint, and passing through the axilla fractured in its course quite extensively the scapula and came out near the spine. The axillary artery or some of its larger branches was wounded. He suffered much from loss of blood, shock to the system, and exposure on the field, previous to his removal to the military hospital at Winchester, Va. While in the hospital, he lost much blood from repeated hemorrhages, and had chills and fever. He reached his home twelve days after the wound was received. During the next ten days he seemed to improve in strength and health. On the twenty-third day after the wound, he had a sudden and profuse hemorrhage, losing a large quantity of blood. I was called by telegram, and when I arrived, found him pale, exsanguineous, pulse 130, and the wound swollen and unhealthy. As the only surgical means available, I decided to ligate the subclavian artery. The operation was performed by candle-light, in the presence of several medical gentlemen. My incisions were made as in the other operation; the external jugular vein and the superficial arteries were ligated; dividing the deep cervical fascia, two larger branches had to be tied. A large nerve, a branch of the axillary plexus, situated over and resting on the artery, was uplifted by every pulsation. On casting a ligature under it and holding it to one side, the artery was found directly beneath, and was ligated between the scaleni muscles. The anterior scalenus had to be divided near the point where it is crossed by the phrenic nerve, which was carefully preserved. The pulsation and hemorrhage ceased immediately, and the patient lost but little blood during the operation. Everything promised a favorable result during the first twenty-four hours. He rested well, took sufficient nourishment, and there was a manifest improvement in the pulse and general condition. Forty-eight hours later he had a severe chill, and on my second visit I found the gun-shot wound in a gangrenous state and the patient rapidly sinking. He died on the twenty-sixth day after the wound was received. The operation was successful as regards the suppression of the hemorrhage and preventing an immediately fatal result. The condition of the patient and the exposure on the field and while traveling had a controlling influence in the final termination of the case.

In June, 1872, W. N., of Warren county, N. Y., was rowing a boat on Cedar River, and getting into the current, was in danger of going over the falls. While using extraordinary effort to avoid the danger, he felt a sharp pain in the axilla, and a sensation as if something had been torn. A swelling soon followed, attended with throbbing pain and inability to move the limb. The tumor increased quite rapidly, carrying the shoulder upward, and protruding forward under the pectoral muscles. He was placed under the care of Dr. McNutt, on the eighth of March, who immediately brought him to Albany. When he arrived he was greatly reduced by the suffering and fatigue of the journey. For several weeks the pain had been severe, preventing sleep and exhausting his strength. The pulsation was strongly expansive over the greater part of the tumor. The operation was performed on the tenth of March, in the amphitheatre of the hospital, the medical and surgical staff of the house and other members of the profession being present.

An incision three and one-half inches in length was made over the center of the clavicle. The superficial and deep cervical fascia and the clavicular portion of the mastoid muscle were divided. But one superficial artery required the ligature. The external jugular was not divided. The artery was reached under the anterior scalenus muscle, which was detached from the rib three-fourths of an inch, to uncover the artery. The deep transverse cervical artery was given off from the subclavian, close to the border of the scalenus, which rendered the division of that artery necessary to reach the subclavian. The ligature was passed from within outwards. Pressure was applied, and the tumor ceased to pulsate. The knot was then secured. The transverse cervical was then tied half an inch from its origin. Only three ligatures were applied, and the patient lost hardly a spoonful of blood. The wound healed kindly. The ligature came away on the fourteenth day. The tumor has gradually diminished, and the sensation and power of motion of the limb, which a week previous to the operation were wanting, have been gradually restored.

At the annual meeting of the American Medical Association, held at New York in 1866, a commitee, of which I was one, was appointed to report on the subject of "Ligature of the Subclavian Artery." The report was made under the direction of Dr. Willard Parker, the statistics being collected by Dr. Wynkoop. At that time 196 cases of ligation of the artery had been reported; 107

died and 88 recovered. The mean time for separation of the ligature was the twenty-first day, the shortest time the eighth day and the longest the one hundred and thirteenth. The subclavian has been tied in its first division thirteen times without one recovery; in its second division, nine times, with four recoveries; in the third, one hundred and seventy-four times, with eighty-three recoveries. Out of sixty-seven cases, twenty-nine have died of hemorrhage. Dr. Rork reports one hundred and eighty-five cases of ligature of the subclavian external to the scaleni muscles, of which one hundred died and eighty two recovered; the result in three cases is not stated. During our late war it was tied seventeen times for hemorrhage, with but two recoveries.

Dr. March said that his father, Dr. Alden, had never had occasion to ligate the subclavian. He had tied the larger arteries forty-three times, among which were the external iliac, common carotid, etc.

Dr. Levi Moore read the following paper:

Some years since I read a paper before this society on the subject of Syphilis. I then confined my remarks more especially to the disease in its primary form, its character, its distinctive features and effects upon the human body.

I have been deeply impressed with the importance of this subject both as it relates to the victim whose vices self-impose this disease and to that larger class to whom this disease is transmitted, hereditary or otherwise.

It is a lamentable fact that by far the greater number of those who suffer either directly or indirectly from syphilis are in no way the authors of their suffering. We are all so frequently brought in contact with this disease, in its primary form and at that period too when it is impossible to prevent the constitutional taint, that we do not wonder at the bitter fruit it bears, involving in a common calamity the innocent as well as the guilty.

Several years ago Mr. N—— placed himself under my care, who was at the time, and had for a considerable period been, suffering from a chancre on the glans penis. The case was complicated by phymosis. Cleanliness, the use of washes, the internal administration of mercurials in the form of pill-hydrarg, and afterwards of proto-iodide of mercury with iodide ot potassium, finally effected an apparent cure. The remedies were continued for a long time, and it was hoped that but little, if any, of the virus of the disease remained in the system of the patient, when they were discontinued.

Afterwards he married, and his wife had a miscarriage at the end of three months' pregnancy. About the same time a troublesome ulcer, syphilitic in appearance and of large size, appeared upon his leg, which only disappeared after long and persistent use of the remedies mentioned above. A second pregnancy occurred, and this time, at the end of seven months, his wife was delivered of a living child covered with a syphilitic eruption, and presenting most unmistakable evidences of constitutional taint. The child lived about one month and died. The mother, to all appearance, retains good health, and yet we need not doubt but that sooner or later she will suffer from the effects of syphilis introduced through her common circulation with that of her unborn child.

Another case is that of a young man whom I was attending for secondary syphilis, who had the characteristic ulcers about the mouth and fauces, and was at time calling upon a young lady of good family and character. Somewhat more than a year afterwards I was called to prescribe for an eruption on the body of the lady, and was surprised to find it syphilitic; also to find a syphilitic sore throat. I then learned that, a year before, she had suffered from a troublesome ulcer on her lip, which was long in healing.

I have introduced these cases only because they are pertinent to some further observations I have to make on this subject. They are by no means exceptional cases. I can recall many analogous ones, but these will suffice my purpose.

Frequent miscarriages result from the effects of this disease, and whenever the offspring is born alive both mother and child are involved in one common misfortune.

If, then, the results of this malady are so unfortunate, it may be asked, Does not the medical art afford a remedy? Cannot this disease be entirely eradicated from the system? There are, I believe, grave doubts in the minds of careful observers whether this disease, when it has once gained a foothold in the human body, can ever be entirely removed. It is believed it may reappear or bear its legitimate fruits in syphilitic or scrofulous offspring. The recognized difficulties in the way of curing those affected with this disease led to the trial of the principle of inoculation, in a Swedish hospital. Patients affected with syphilis were repeatedly vaccinated with syphilis virus till a constitutional tolerance had been established and the virus ceased to produce any effect. Many cases were subjected to this treatment, and satisfactory results claimed. Although several years have elapsed since I read the statement, I have not learned that any other hospital has seen fit to continue the experiment.

I will next direct the attention of this society to a sanitarian view of this disease. Multitudes of our young men enter upon married life tainted by this poison; wives and children alike suffer. Others still, unfaithful to their marital relations, contract this loathsome disease, communicate it to their wives, and doubly entail it upon their children. The importance of this subject has even led to legislation on the matter of prostitution, and the city of St. Louis, and some others, have instituted a sanitary police to regulate prostitution, and in this way to protect the innocent from its evils. The moral as well as the sanitarian aspect of this question has been not a little discussed; and while it has been warmly commended on some hands, it has been bitterly denounced on others. It rests with the medical profession to shape and direct public opinion to the proper view of this question.

My purpose in reading this paper has been accomplished if I have awakened in the mind of any member of this society a deeper interest in this important subject, and a more earnest desire to shield the innocent and unsuspecting from its destructive influences.

Dr. BIGELOW remarked that Dr. Henry, of New York, had recommended the combination of quinine with mercury, having found the exhibition of the two together to anticipate the effects of mercury alone.

Dr. BAILEY said that during the past year he had had sad experiences with some stubborn cases of syphilis. He had used the bichloride of mercury and iodide of potassium, singly and alone. Ricord has said that it is possible to eradicate syphilis entirely from the system, recommending iodide of potassium in heroic doses as a specific. This treatment was tried in a case of mucous patches and warty excrescences of the tongue and fauces, which other treatment did not seem to affect, with the happiest results. He gave it in a simple solution in water, and had not found it irritating to the stomach.

Dr. VAN DERVEER presented two specimens of amputated cervix uteri. He operated for the removal of one of them some time since, and the patient, who up to that time had been sterile, became pregnant three months after, and he had just attended her in a second confinement. There is no rigidity of the os, and the cervix is quite normal.

The second specimen was from an unmarried woman, aged 35, upon whom he operated recently. She had been troubled for ten years by a difficulty in urinating; something, as she said, coming down, had to be pushed back before urine could be passed. There was found longitudinal hypertrophy of the cervix, the organ protruding on standing. For the operation the patient was etherized, the parts exposed by a Sim's speculum, the neck brought down, and a silver wire passed around it. The cervix was then amputated with a curved scissors, one and one-half inches anteriorly and two inches posteriorly. Four sutures were introduced to unite the edges of the mucous membrane. She now menstruates normally and already feels much better. He preferred the scissors to the galvano-cautery, as the latter leaves a cicatrix which can never be covered with epithelium, while by using the scissors the mucous membrane can be united by sutures and so be made continuous. There is little hemorrhage by this method. The wire acts as a tourniquet, and also aids by bringing the organ into position convenient for operation.

---

SEMI-MONTHLY MEETING, April 23, 1873.

The Society met at the usual place and hour, and was called to order by the President, Dr. VAN DERVEER.

After the minutes of the last meeting had been read and approved,

Dr. A. P. TEN EYCK read a paper on Phlegmasia Dolens. This is a comparatively rare disease, yet one whose causes, symptoms, pathology, and treatment, it is very important the practitioner should be thoroughly acquainted with. It is most common in women after parturition, but is not confined to such, nor to the female sex even: it may occur during pregnancy, in non-pregnant women and in men. Druitt gives two cases of the disease in men. Still it may be classed among the diseases peculiar to women. Inflammation of the femoral vein is the essence of this disease, the origin of which is referred, in parturient women, to the various influences brought to bear in connection with the process of child-birth, though frequently it is impossible to find any cause. Various views have been held, however, as to its pathology. The older writers supposed it to be due to a deposit of milk in the affected limb, led, no doubt, to this view by its peculiar white color and by the fact that the lacteal secretion is much diminished upon the occurrence of this disease. The name *milk leg*, though arising from an absurd view of its pathology, is not inappropriate in view of its white appearance.

The following case was given: —

Mrs. R., æt. 24, was a woman of good constitution, medium size and fair complexion. She was married in 1869, became pregnant soon after, and aborted at the third month, from a fall. She again became pregnant, and was delivered, naturally, in November 1872. The labor was entirely normal and easy. No untoward symptoms presented for two weeks after, during which time she kept her bed, excepting a persistent headache which begun on the day following delivery. On the 13th day she complained of pain in the left groin, hip, and calf of the leg, which was not preceded by chill or any febrile action. This pain increased, the patient became restless, appetite impaired, tongue slightly coated, pulse 120 on the 7th day from the outset, and the limb became much swollen. These symptoms gradually grew worse: the lacteal secretion diminished in amount until it was entirely suspended; the lochial discharge continuing about the same; the pulse ran up to 140; the pain was excruciating and the limb swollen throughout, and presented the characteristic white, shining look. The femoral vein could be felt "rolling under the finger like a cord." The bowels were slightly constipated, the skin moist, the tongue not very much coated. The pulse never fell below 140 until the middle of January, when the acuteness of the symptoms diminished. The relief, however, was of but short duration, for the symptoms returned very suddenly, this time the other limb being affected. The pulse rose to 160, and all the previous symptoms became very acute. The limb first affected returned to a normal condition. The pulse did not fall below 130 until the 1st of March, when the disease gradually became less acute. About this time an eruption resembling small-pox came out on the limb and lasted two weeks, the spaces between the pustules being of a scarlet color. In four months from the onset she was able, for the first time, to walk across the room unassisted; and at this time (the last of March) she can walk about the house, but soon becomes tired. The feet are still swollen, and pain is felt in the limbs.

The treatment has been supporting and anodyne, which seemed to be the main indications in the case. Acetate of potash was given as a diuretic, with a view to lessen the œdema. Local applications were made use of to relieve the pain and inflammation. To the first limb a weak solution of carbolic acid was kept applied, as hot as the patient could bear it, which seemed to give great relief. The second limb was treated according to a plan recommended by Dr. Crichton in a paper read before the British Medical Association in 1871. This consisted in applying a solution (30 grs. to the ounce) of the sulphate of iron, as hot as could be borne. This was followed by marked diminution of the pain and swelling, and was recommended as the best local application. The limb was also bandaged from the toes to the body, which was believed to be a valuable part of the treatment.

Dr. E. H. Davis said that he had seen a large number of cases of plegmasia dolens. He was not prepared to say that it was not a disease of the femoral vein; but with his ideas of what constitutes phlebitis, he did not think that his cases presented symptoms consistent with this view of its pathology. A large majority of his patients complained first of pain in the calf of the leg, and not in the femoral region. This pain was not aggravated by pressure, nor could anything abnormal be detected by touch, but exertion of the muscles, as in pressing the heel against an object, increased the pain. Spreading upwards, however, it has followed the course of the lymphatics and femoral vein. By some it is thought to be a general inflammation of the tissues of the limb,—the veins, lymphatics, cellular and muscular tissues. Its pathology may be regarded as still unsettled. He had known it to occur in the non-puerperal state; and had observed it quite as often, if not generally, in those previously strong and vigorous. A case was mentioned of a robust young woman in the country, who, after exerting herself vigorously while berrying, during the time being exposed to rain, was taken with pain in the calf of the leg, and the disease pursued the course of phlegmasia dolens. In regard to treatment, he endorsed the use of warm applications locally. He applied it by means of bags of hops dipped in warm water. Sulphate of iron he had never tried. The general treatment depends on the condition of the patient, for it does not follow that because we have a certain disease to deal with we must necessarily use sustaining remedies. These are to be used according to the indication. Occurring in a plethoric person, he would bleed early. In the later stage of the affection stimulating embrocations were recommended.

Dr. HALE gave an account of a case which he regarded as, and which presented marked symptoms of phlegmasia dolens, occurring in a man. The patient had been attended by a number of different physicians, and various diagnoses made, some regarding it as periostitis, which seemed the more probable in the later stage of its course, as fistulæ developed about the lower end of the femur. He died about a year after the disease set in.

Dr. J. S. BAILEY remarked that among the first seventy-five cases of midwifery which he had attended in 1871, four cases of phlegmasia dolens occurred. In three, it appeared in one leg only; one, in whom both legs were attacked, had borne twins. She died in three months from exhaustion. One case was a primipara, the others multiparæ. The treatment adopted was supporting and anodyne, the limb being wrapped in flannel clothes, wrung from hot water, applied as hot as could be borne, and covered with oiled-silk. This was continued till the inflammation subsided. The eruption on the limbs spoken of by Dr. Ten Eyck, he thought was produced by the applications used.

Dr. FREEMAN thought that the disease is more liable to occur in those of lymphatic temperament and light complexion. In a recent case he had directed turpentine stupes to be applied to the limb; the turpentine was applied so freely by the nurse that it produced vesication. Warm fermentations were then used, and the patient made an unusually rapid recovery, the attack lasting only three weeks. He thought the speedy cure was due much to the vesication.

Dr. CURTIS remarked that Dr. T. G. Thomas, of New York, recommended the application of a blister along the course of the vein, placing it to one side so as to leave the vein free.

Dr. CRAIG expressed the opinion that the source of various inflammatory conditions occurring during the first week or so after confinement was in the uterus. He had no doubt but that a general phlebitic condition of the uterus itself was first set up, which may extend to the peritoneum, lower limbs, or elsewhere. He would base his treatment on this view of the pathological condition, and begin by treating the womb and vagina, irrigating the parts with a solution of carbolic acid, &c.

Dr. DEVOL said that he did not wonder that these conditions were induced when we consider the compression the soft parts receive by the passage of a large child's head. He thought the inflammation was not necessarily confined to the veins, but involved also the lymphatics and other tissues. He endorsed the local treatment spoken of. In some cases he had immersed the limb in brewer's yeast with good effect.

Dr. JOSEPH H. BLATNER read a paper entitled, "Some late facts concerning the Cervix uteri during Conception." He stated that the investigation of the question as to how the semen of the male is brought into contact with the ovula of the female, or in other words, the process of conception, involves the consideration of the motive powers which propel the spermatic fluid, the part which the cervix takes, and many other intimately connected questions.

As to how the spermatic fluid is propelled through the cervical canal, opinions vary; the following named forces have been given:

1. Ejaculation during coition; 2. Peristaltic action of the vaginal canal; 3. The power of absorption by the uterus; 4. The piston-like action of the penis; 5. Capillarity; 6. Ciliary motion of the cervical epithelium; 7. Self-movement of spermatozoa.

The theory that the sperm is thrown directly into the uterine cavity by ejaculation was advocated by Holst, one argument being that there could be no other reason for the existence of the ejaculation. It is thought, however, that this force is necessary to carry the fluid through the long urinary canal and the vagina; besides it is observed in animals that deposit their sperm on ready laid eggs. The force cannot be strong enough to carry the fluid into the uterus; and the meatus urethræ of the male does not coincide with the os externum, as is shown most clearly in the dog. The existence of valve-like or spiral folds, seen to move, particularly in the sheep and sow, offers an obstacle that can hardly be overcome by an injected stream. The additional theory that the canal of the cervix is open during coition, either by erection (Holst) or by reflex muscular action (Vievordt), is opposed by the fact of the close apposition of the walls and the compact structure of the organ.

The peristaltic action of the vagina was observed by Kehrer in animals This may exist, but cannot alone act with sufficient force to carry the fluid through the cervical canal.

The absorbing power of the uterus, either by erection or by peristaltic action of the organ, has been asserted, but was not considered proven. Ducilliez and Kuss advocated this theory, holding to the view of its being effected by erection of the uterus — erection causing the formation of a cavity, and thus producing a vacuum. Objections are, that the mechanism of closure of the uterus, like that which is produced in the sucking mouth by means of the pharyngeal muscles, is not in accordance with our knowledge of the uterine muscles; no direct proof has been established either of erection or of peristaltic motion of the uterus; no such problematic force is necessary.

To prove that the seminal fluid is forced into the uterus by the piston-like action of the penis, it is necessary to show that the vagina is inflexible, has no pocket-like vault, and that it encases the penis so closely that the fluid is allowed no other outlet than the cervical canal; all of which is known not to be the case.

The theory of capillary attraction was entertained by Spath and others. The relatively small lumina and thick walls of the uterus are well adapted to develop capillary force, but these lumina are already filled with a fluid of almost exactly similar consistence as the spermatic.

As to the spermatozoa being carried forward by ciliary motion, the ciliæl of the cervix and fallopian tubes move in an opposite direction to that which these pursue.

Self-movement of the spermatozoa was thought to account for their passage into the uterine cavity, other motive factors being perhaps auxilliary. The passage of the spermatic fluid *in toto* into the uterus is not proven, nor is it necessary. Dr. Blatner made experiments which showed the possibility of the emigration of these cells from their fluid; of their retaining their motive power in different fluids; and that this capacity is variable according to the nature of the fluid surrounding them. This migratory action has analogies in other cells. Direct experiment by Lott, on a bitch in heat, demonstrated the capacity of these cells to move along the canal unaided. Examination of animals or man shortly after ejaculation has taken place shows spermatozoa only in the vagina or beginning of the cervix; the observations of Sims and others, showing their existence higher up thus early, are not to be considered conclusive. Cases of conception without penetration show the possibility of spermatozoa moving from the vulva into the uterus. That such cases have occurred cannot be doubted. The acid secretion of the vagina is not favorable to their vitality, but this is neutralized near the os by the cervical secretion. The conditions then favoring impregnation are the deposit of the semen at the os externum, or within the lower part of the cervical canal, which is possible by the pressure of the penis and the force of ejaculation, aided, according to Sims, by the pressure of the cervix against the glans by the contraction of the superior constrictor vaginæ, and by the dipping of the cervix into the pool of semen in the vagina. Under these circumstances, it was believed the spermatozoa could most easily move to their destination.

